# A Case of Clozapine-Induced Myocarditis: Navigating the Risks of the Gold Standard Treatment

**DOI:** 10.1192/bjo.2025.10702

**Published:** 2025-06-20

**Authors:** Srishti Agarwal, Nitish Jayanth Kumaraswamy, Manicavasakar Kathirgamar

**Affiliations:** Central and North West London NHS Foundation Trust, London, United Kingdom

## Abstract

**Aims:** We present a case of a 36-year-old patient on clozapine who was an inpatient in a mental health rehabilitation service diagnosed with Schizophrenia. During clozapine titration, the patient complained of chest pain and elevated temperature. The patient was subsequently transferred to the emergency department for further evaluation and management. Following the discontinuation of clozapine and the initiation of brief supportive medical therapy, the patient’s symptoms, ECG changes, and troponin levels fully resolved.

**Methods:** A 36-year-old Asian male diagnosed with paranoid schizophrenia, admitted to rehabilitation psychiatry. With no significant past medical history, clozapine was introduced in January 2024 using a slow titration protocol, resulting in minimal mental state improvement and physical deterioration. In February 2024, he developed a severe chest infection requiring a two-week hospitalization, during which clozapine was discontinued.

After discharge, clozapine was reinitiated with a slow titration protocol; however, in March 2024, the patient presented with tachycardia, elevated temperature, and chest discomfort during the titration process. ECG findings revealed sinus tachycardia with a heart rate of 112 bpm, a prolonged QTc interval of 470 ms, frequent premature ventricular complexes (PVCs), and ventricular bigeminy. Blood tests showed an elevated troponin T of 16 ng/L (normal range: 0–14). During an A&E evaluation, the patient was asymptomatic apart from the noted ECG abnormalities and was discharged within 24 hours with recommendations to reassess his medications. Clozapine was subsequently discontinued, and no antipsychotic therapy was initiated immediately.

A follow-up ECG performed one week later showed normalized findings, including a heart rate of 54 bpm and a QTc interval of 443 ms, while troponin T levels returned to normal at 14 ng/L. Clinical resolution of myocarditis-like symptoms was observed.

**Results:** In this case, the patient developed significant ECG abnormalities and elevated troponin levels within two months of clozapine initiation. These findings, combined with clinical symptoms, necessitated immediate discontinuation of clozapine. Subsequent resolution of cardiac abnormalities within one week strongly indicated clozapine-induced myocarditis or cardiotoxicity. This outcome aligns with existing evidence that supports stopping clozapine in the presence of significant cardiac derangements.

**Conclusion:** This case emphasizes the critical need for monitoring for any adverse effects from clozapine particularly in the titration phase. Regular monitoring, including ECG and blood tests, is essential to identify early signs of myocarditis or cardiotoxicity. If there are any symptoms indicating cardiac abnormalities, clozapine should be discontinued immediately and referral should be made to medical or cardiology specialist for further evaluation.

